# Supplementary figures and images for: Simulations of dynamically cross-linked actin networks: Morphology, rheology, and hydrodynamic interactions
Source: PLoS Comput Biol. 2021 Dec 6;17(12):e1009240. doi: 10.1371/journal.pcbi.1009240 (PMC8675935; doi:10.1371/journal.pcbi.1009240)

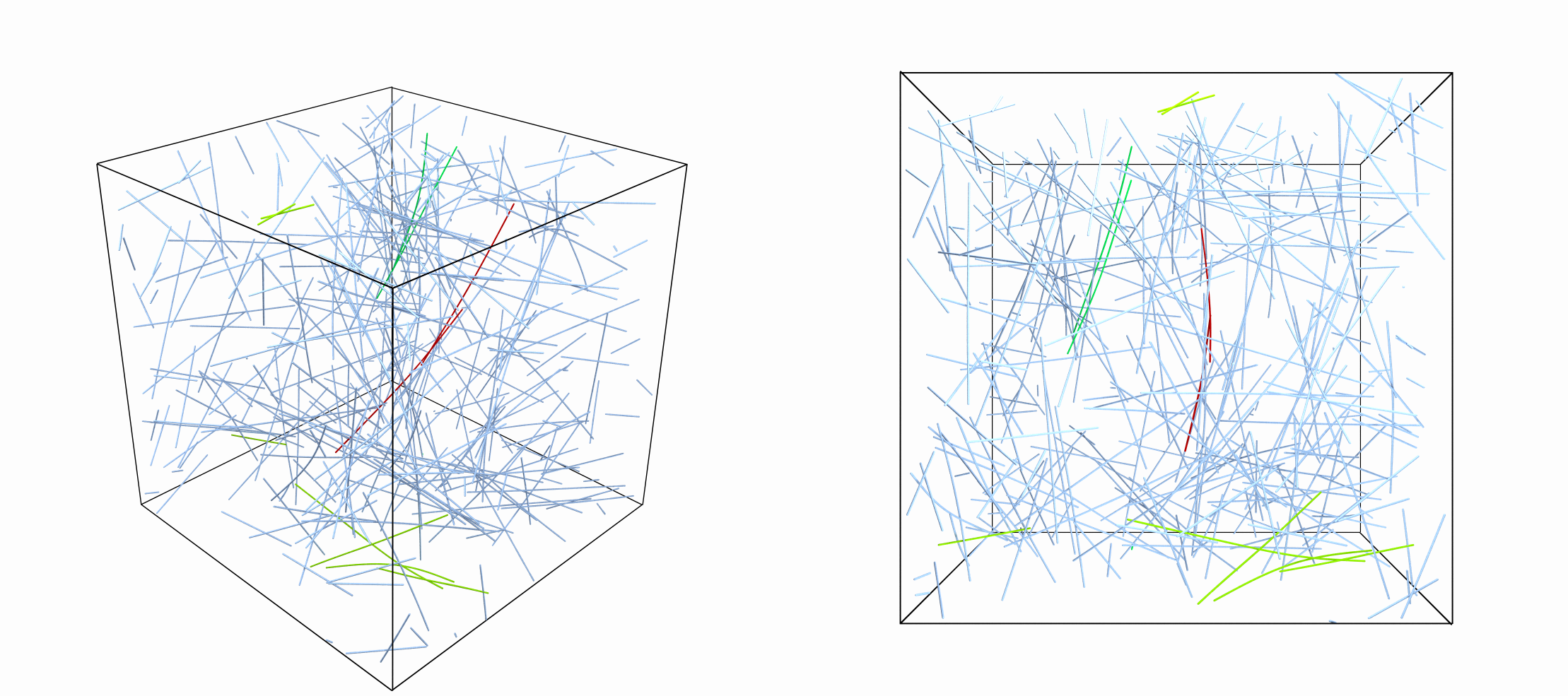

Supplement: S3 Fig — Side (left) and top (right) view of the homogeneous meshwork with κ = 0.007 pN ⋅ μm2, which is 1/10 of the stiffness of actin. Despite the smaller bending stiffness, the fibers still appear relatively straight, with the exception of some of the fibers in bundles. (TIFF) [file pcbi.1009240.s003.tiff]
